# Diagnosis of Latent Tuberculosis in Patients with Systemic Lupus Erythematosus: T.SPOT.TB *versus* Tuberculin Skin Test

**DOI:** 10.1155/2014/291031

**Published:** 2014-05-28

**Authors:** Maria Del Mar Arenas Miras, Carmen Hidalgo-Tenorio, Pilar Jimenez-Gamiz, Juan Jiménez-Alonso

**Affiliations:** ^1^Department of Internal Medicine, University Hospital Virgen de las Nieves, Avenida de las Fuerzas Armadas No. 2, 18004 Granada, Spain; ^2^Department of Infectious Diseases, University Hospital Virgen de las Nieves, Avenida de las Fuerzas Armadas No. 2, 18004 Granada, Spain; ^3^Department of Clinical Analysis, University Hospital Virgen de las Nieves, Avenida de las Fuerzas Armadas No. 2, 18004 Granada, Spain

## Abstract

Early studies in patients with systemic lupus erythematosus (SLE) reported increased incidence of tuberculosis. The tuberculin skin test (TST) is the technique of choice to detect latent tuberculosis infection (LTBI) but has several limitations. *Objectives*. We compared TST and the newer T.SPOT.TB test to diagnose LTBI in SLE patients. *Methods*. In this observational cohort study conducted between August 2009 and February 2012, we recruited 92 patients from those attending the SLE clinic of our university hospital. Data recorded were epidemiological and sociodemographic characteristics. Laboratory analyses included TST and T.SPOT.TB tests. *Results*. Of the patients studied, 92% were women with an average age of 42.7 years. Overall, the degree of correlation between the two tests was low (Kappa index = 0.324) but was better in patients not receiving corticosteroids (CTC)/immunosuppressive (IS) therapy (Kappa = 0.436) and in those receiving hydroxychloroquine (Kappa = 0.473). While TST results were adversely affected by those receiving CTC and/or IS drugs (*P* = 0.021), the T.SPOT.TB results were not. *Conclusion*. Although the TST test remains a useful tool for diagnosing LTBI in SLE patients, the T.SPOT.TB test is perhaps better employed when the patient is receiving CTC and/or IS drugs.

## 1. Introduction

SLE is an autoimmune disease of unknown aetiology, which can affect any organ and system [1]. Due in part to this and the IS treatment administered, the patients with SLE have a high risk of acquired infections, which constitute one of the principal causes of death in this group of patients [2, 3]. To date, there have been several studies published on subjects with SLE that have shown an increased incidence of tuberculosis (TB) in the lung and nonlung tissue, compared to the general population [4–12]. Among the different risk factors implicated in the development of TB is the use of CTC. Hence, it is recommended that the diagnosis of LTBI is made, even in the general population, before initiating treatment [13].

The Mantoux test (or the TST tuberculin skin test or the purified protein derivative (PPD)) remains the classical technique in the detection of LTBI but has several limitations including the higher probability of false negatives in immune-compromised patients and, as well, false positives not only in those vaccinated with BCG (*Bacillus* Calmette-Guérin) but also in those who had had a previous infection with nontuberculosis* Mycobacterium* [14].

Newer techniques of LTBI detection, based on the determination of interferon gamma release assays (IGRAs), have been used in different types of patients and different geographic areas in order to evaluate their usefulness. According to a meta-analysis and systematic review of the recent literature [15], the calculated specificity of T.SPOT.TB in the diagnosis of LTBI was approximately 98% (95% CI: 86.8 to 99.9%) and 89% for the TST (95% CI: 84.6 to 92%). But this meta-analysis had some limitations, including a low number of studies evaluated in calculating the specificity of the IGRAs. In another meta-analysis published earlier in the nonvaccinated population [16], the sensitivity of T.SPOT.TB was 90% (95% CI: 86 to 93%) and 77% for the TST (95% CI: 71 to 82%). The sensitivity was calculated based on studies composed of patients with confirmed TB, and the conclusion was that the measurement of T.SPOT.TB had greater sensitivity than Quantiferon-TB Gold (QTF-2G) which was indicated as being more useful in immune-compromised patients.

To date, there have been only 2 articles comparing QTF-2G [17, 18] with TST for the diagnosis of LTBI in patients with SLE. The inconvenience of both studies is that they were performed in areas where vaccination with BCG was already in effect. This limits the extrapolation of the data to our country where it has not been recommended by the majority of the autonomous governments of several regions of Spain [14]. There have not been comparisons between the efficacy of IGRAs such as Quantiferon-TB Gold In-Tube (QTF-3G) or the T.SPOT.TB* versus* TST. There is no information available on the patients being treated for LTBI based on the results obtained or the usefulness of the new IGRAs in standard clinical practice. Finally, there are no studies in our geographical area of Europe (i.e., Spain) that evaluated the usefulness of IGRAs in patients with SLE.

Hence, we proposed analysing, in patients with SLE falling within our remit of healthcare provision, the concordance between T.SPOT.TB and TST in the diagnosis of LTBI. The secondary objective was to generate a protocol for the diagnosis of LTBI in these patients.

## 2. Patients and Methods

The study was cross-sectional, observational between August 2009 and February 2012. Following written informed consent, 92 patients with SLE were recruited from those attending the Clinic of the Systemic Autoimmune Disease of the* Hospital Universitario Virgen de las Nieves *(Granada, Spain). The patients needed to have fulfilled 4 or more diagnostic criteria of the American College of Rheumatology (ACR). Those patients <18 years of age and those judged to be mentally unable to provide independent consent had the consent obtained from the parents or guardians. The study was approved by the ethics committee of the hospital and the data were coded to maintain anonymity.

At the baseline clinical visit, a personal history was taken. Information sought included zone of residence, risk factors for TBL (including profession, contact history, and family status) BCG vaccination, age, gender, months since diagnosis of the disease (disease duration), other associated immunosuppressant diseases, current treatment for SLE, history of TST, or previous treatment for LTBI. Laboratory tests performed included full blood screening, urine analysis, antinuclear antibody (ANA), C3, C4, lymphocyte populations, TST and booster (to the patients initially nonresponsive to TST and repeated within 7–20 days), T.SPOT.TB, and chest X-ray. The Systemic Lupus Erythematosus Disease Activity Index (SLEDAI) and Systemic Lupus International Collaborating Clinics (SLICC) organ damage index were determined. Patients diagnosed with LTBI, and for whom treatment was indicated [19], had the appropriate treatment initiated, provided existing active TB was not present.

### 2.1. Definition of Variables

- TST was considered positive according to the criteria of the American Thoracic Society [19] when >5 mm and the patient was receiving IS treatment or >15 mg prednisone for >1 month or >10 mm in the rest of the cases.
- T.SPOT.TB positive, negative, or indeterminate were according to the criteria of our laboratory, using standard techniques (Oxford Immunotec, Oxford, UK). A typical result would be expected to have few or no spots in the Nil control and >20 spots in the Positive Control. In cases where the negative (Nil) control had ≤10 spots, the result was defined as positive if Panel A-Nil and/or Panel B-Nil had ≥8 spots. If the Nil control had >10 spots or Positive Control had <20 spots, the result was considered indeterminate. If the above criteria were not met, the result was defined as negative. (Available at http://www.oxfordimmunotec.com/USpageInsert.)
- Patients were considered immunocompromised if receiving treatment with the following drugs: mycophenolate, methotrexate, tacrolimus, leflunomide, azathioprine, cyclophosphamide, and/or CTC at whatever dose.
- The two tests were considered concordant when the same results were obtained for both of them.
- The diagnosis of TBL was considered when any of the tests were positive (TST or T.SPOT.TB).
- Prednisone dose was considered physiologic at <7.5 mg/day [13].
- Normal levels of dsDNA according to our local laboratory values were 0–30 UI/mL.

## 3. Materials and Methods

The TST was performed with an injection in the ventral surface of the forearm, of 0.1 mL PPD (variant RT-23), at a dose of 5 UT; the result is to be read within 72 hours. The TST was performed by trained personnel.

The IGRA technique used was the T.SPOT.TB (Oxford Immunotec) which is a technique that counts the T effector cells that respond to stimulation by antigens of* Mycobacterium tuberculosis* (ESAT-6 and CFP10). The technique was applied and monitored by qualified personnel of the Clinical Analysis Laboratory of our hospital.

### 3.1. Statistical Analyses

Descriptive analyses of the principal variables included calculated means and standard deviation for the quantitative variables and absolute and relative frequencies for the qualitative variables. Bivariate analyses were performed to evaluate the variables associated with the diagnosis of LTBI with the two tests employed (TST and T.SPOT.TB). Quantitative variables following a normal distribution were analysed with Student's* t*-test or the Mann-Whitney test for those variables nonnormally distributed. The qualitative variables were analysed with Pearson's *χ* ^2^ test or the Fisher test. Significance level was set at *P* < 0.05.

The degree of concordance between the two tests was determined with the Kappa index. The results of the tests were evaluated using the classification of Landis and Koch in which a value of *κ* < 0.20 would be poor, 0.21–0.40 weak, 0.41–0.60 moderate, 0.61–0.80 good, and 0.81–1.00 very good agreement.

The diagnostic precision of the study was measured as the total accuracy value.

The SPSS statistics package (version 19) was used throughout.

## 4. Results

### 4.1. Description of the Patient Cohort; Results of TST and T.SPOT.TB

92 consecutive patients were included in the study with SLE, of whom 92% were female. The mean age was 42.7 years (range: 14–77 years). The demographic, clinical, and laboratory variables are summarised in Table 1.

Of the 92 patients, the T.SPOT.TB was positive in 5 (5%), indeterminate in 4 (4%), and negative in 83 (90%). The TST was positive in 6 patients (7%) and negative in 86 (94%) (Table 2). Positive LTBI (whether with TST or with T.SPOT.TB) was diagnosed in 9 patients (10%). As such, the prevalence of LTB in our SLE patients in the study was 10%.

Diagnostic precision or efficiency (*total accuracy*) of the evaluation was 92%.

The degree of concordance between the two tests in the overall study population was low, according to the Kappa index (*κ* = 0.324). When this concordance was analysed only in those patients not treated with CTC or IS drugs, the values improved (*κ* = 0.436), as well as in those receiving hydroxychloroquine (*κ* = 0.473) (Table 3).

During the period of study, we diagnosed 9 patients with LTBI. We did not identify any patients with active TB. There were 3 patients (33%) who received treatment for LTBI, of whom 2 (22%) needed to have their medication suspended because of digestive intolerance, nausea, and epigastric discomfort. No severe adverse effects of grades III-IV was recorded. Of the patients diagnosed as having LTBI (*n* = 9), 1 (11%) did not wish to receive treatment, 2 (22%) were lost to the study having moved out of the area, and 3 (33%) did not begin treatment due to decision by the attending physician, one for having active chronic liver disease due to HCV and another due to being T.SPOT.TB negative. One patient was TST positive, without any personal history of risk or X-ray findings of fibrotic tracts suggestive of prior infection. These 3 patients had not been receiving IS treatment or CTC for several years.

### 4.2. Univariate Analyses

Of the patients, 64% were receiving CTC or other IS drugs; 24% received CTC alone, and 40% received both. Comparing the CTC-alone group with the combination therapy group, the latter had greater organ damage (*P* = 0.05) and were predominantly women (*P* = 0.023) but with no statistically significant differences with respect to TST or T.SPOT.TB. We did not find significant differences between those patients receiving daily doses of prednisone, above and below 7.5 mg dose. As such, we considered only two groups in the statistical analyses, that is, those with and those without IS treatment.

The results of TST were affected in patients receiving CTC and/or IS; that is, in this group of patients there was a greater number of TST negative, with only 17% of cases being positive (OR: 10.30; 95% CI: 0.011–0.866; *P* = 0.02). Further, the patients with TST negative had been receiving IS (*P* = 0.048) and CTC (*P* = 0.008) treatment for a longer period of time. The rest of the variables analysed did not significantly influence the TST outcome (Table 4).

The results of T.SPOT.TB were not affected by IS (except for a prolonged treatment with mycophenolate) or CTC. However, age had a significant influence; that is, older patients were diagnosed with LTBI in more occasions with T.SPOT.TB than with TST (*P* = 0.002) (Table 5). Conversely, we found that having an initial positive TST was associated with a greater probability of T.SPOT.TB being positive (*P* = 0.033). Indeterminate T.SPOT.TB results were related to a longer time to diagnosis (duration) of the disease (*P* = 0.028) and SLICC organ damage index (*P* = 0.002) (Table 6).

There were no statistically significant associations between TST/T.SPOT.TB results and IS therapies such as tacrolimus (*P* = 0.71/*P* = 0.73), leflunomide (*P* = 0.68/*P* = 0.71), azathioprine (*P* = 0.57/*P* = 0.60), and cyclophosphamide (*P* = 0.79/*P* = 0.81).

Finally, we observed that the patients receiving hydroxychloroquine had a higher grade of concordance between the two tests (*P* = 0.007).

## 5. Discussion

Tuberculosis is an important public health problem worldwide. In the European Union (EU) it continues to be an unresolved issue, with considerable differences between countries and, over the past few years, the rates of multiresistant infections have increased [20]. Overall levels within the EU are improving. However, despite known underreporting in Spain, there are considerable differences between autonomous regions of Spain with respect to control of the disease [21].

The prevalence of LTB in our study was 10%, which coincides with the percentage of patients with risk factors for tuberculosis (9.8%). Our study was conducted in a zone considered low with respect to incidence of TB within Europe [22] and represents the first study of its kind in a nonvaccinated population of SLE patients.

In our group of patients with SLE, CTC (irrespective of the dose) and other IS drugs negatively affect the results of the TST, which results in an underdiagnosis of the disease when only the TST test is employed. We have observed this event principally with CTC, mycophenolate, and methotrexate, these patients having a 10-fold higher probability of a negative TST. No statistically significant differences with other IS drugs (tacrolimus, leflunomide, azathioprine, and cyclophosphamide) were noted, probably due to the limited number of patients in the study. The use of CTC can cause anergy at low doses due to the alterations that are produced, principally, on cellular immunity and including, in isolated cases, humoral immunity [23]. On the other hand our results showed that positive TST was correlated with positive T.SPOT.TB, indicating the reliability of the TST. The test continues to be the test of choice for LTBI detection in patients with non-IS medication-related lupus. Our results suggest that T.SPOT.TB could be the diagnostic tool of choice for diagnosis of LTBI in patients with IS and also demonstrated greater usefulness than TST in older patients. These results need to be confirmed in further studies with a higher number of SLE patients selected from a geographic area with an incidence of tuberculosis similar to ours.

In studies published to date, there has been an increase in indeterminate T.SPOT.TB results in patients with SLE receiving IS [24]. The percentage of indeterminate values in our study was 4.3% and was similar to the 2.5% observed in the study by Yilmaz et al. [17] but much lower than the 32.4% observed by Takeda et al. [18]. This high value was considered to have resulted from the high levels of SLEDAI, lymphopenia, and the presence of the disease. In our series of patients, the percentage of indeterminate values was related to the greater time since diagnosis (duration of the disease) and higher levels of SLICC. However, we did not observe association with the activity of the disease despite having a homogeneous population comparable to that described in other studies. This leads us to believe that our cohort was well controlled, with a mean SLEDAI around 3. We did not find association between a high activity and low TST reaction, as had been described earlier by Pascual-Ramos et al. [25] whose study indicated that the inactive-disease patients present greater TST reaction than the active-disease patients. In their study, in contrast to ours, the mean level of SLEDAI was around 7.

In analysing the levels of lymphocyte populations in our patients, we observed that the levels of CD4 and CD8 were maintained stable despite the high percentage of lymphocytopenia recorded (58.1%) and, as such, a response to T.SPOT.TB was possible. In contrast to previous studies in patients with SLE [17, 18] in which an ELISA assay was used, our study employed a technique in which the polymorphonuclear cells were separated from the peripheral blood to guarantee that, in the detection assay, a normalised number of cells (i.e., cells per unit volume) were used; this refinement is more useful in patients with immune systems alterations [26].

CTC use in low and moderate doses results in slight reductions in the T lymphocytes in the peripheral circulation (more CD4 than CD8). The consequence is a delayed hypersensitivity response and unlinked cutaneous anergy [27]. This event could affect the TST result, but the outcomes of the T.SPOT.TB are not affected by cutaneous anergy.

Hydroxychloroquine, widely administered in patients with SLE, has an immune-modulatory effect and, as has been highlighted in other studies, is a protective factor against infections [28]. In our study, this role is highlighted as the concordance between TST and T.SPOT.TB in patients receiving hydroxychloroquine, that is, a higher correlation between the two tests in this group of patients.

The overall concordance between T.SPOT.TB and TST in our patients with SLE was low. These findings are similar to those previously published [17, 18]. However, when the patients are segregated with respect to treatment with IS or CTC, those not receiving this treatment have an improved concordance, an event that needs to be confirmed in further studies. In this aspect, our results are different from those published [17] in which the concordance improved when patients treated with IS and CTC are included in the overall analysis. This could be due to differences between populations studied, for example, vaccination of 97.4% in some studies* versus* only 2.2% in ours. One difficulty with this study is that the use of TST for the diagnosis of LTBI is inappropriate in populations with higher percentage of vaccination (97.4%), due to the number of false positives being higher.

Studies conducted in zones similar to ours in which the prevalence of TB is similar to ours [13] have demonstrated how the treatment with CTC, including that at a dose of 7.5 mg/day, increased the risk of TB. Based on these data, and taking into account that CTC treatment is employed in the great majority of patients with SLE and that many of them have been on treatment over many years, we propose a standard procedure for the outpatient clinic. This focusses on a screening test for LTBI in the evaluation of all patients with a recent diagnosis of SLE. For a diagnostic protocol of LTBI in patients with SLE, many of whom will have been on treatment for several years, we propose the following.

1. For patients without IS or CTC, we would initially perform a TST. If this was positive and there is no history of vaccination, we would treat the LTBI. If the TST was negative, we would administer a booster over two weeks and, in the case of repeated negativity, the diagnosis of LTBI is excluded.
2. In patients receiving CTC or IS we propose to proceed directly to T.SPOT.TB and make clinical decisions based on the results.

One of the principal limitations of our study, and the diagnosis of LTBI, is that there is no “gold standard” test to compare the results. Hence, we need to compare the different techniques employed in each specific population to evaluate the usefulness. Another limitation is the number of patients. Due to the low incidence of TB in our geographic area and the low incidence of SLE in the general population, the number of patients recruited into the study was limited. This limitation would be reduced if the study was multicentred and included geographic areas with incidences of TB similar to ours. However, the multicentred studies carry other limitations too.

## 6. Conclusions

Based on our findings, we conclude that, in patients with SLE who are not on treatment with CTC or other IS drugs, the TST test continues to be a useful technique for the diagnosis of LTBI in our (Spanish) environment. In case the patient is receiving CTC (irrespective of dose) and/or other IS drugs, the result of the TST can be affected, increasing the number of false negatives. In these cases, T.SPOT.TB test would be the diagnostic technique of choice. Neither SLE by itself nor its activity appears to influence the TST result, the IS treatment being responsible for alterations in these results. Finally, in the patient with lupus, greater damage to organs and time of clinical evolution of the disease (disease duration) have a higher risk of indeterminate T.SPOT.TB resulting, perhaps, from deterioration of the cellular immune system.

## Figures and Tables

**Table 1:** Clinical and laboratory characteristics of the SLE patients studied.

Clinical characteristics	
Age, years, mean ± SD	42.71 ± 14.88
Females, *n* (%)	85 (92.4)
SLE diagnosis duration, months (IQR)	132 (60–216)
Risk factor for LTBI, *n* (%)	9 (9.8)
BCG vaccinated, *n* (%)	
Nonvaccinated	90 (97.8)
Vaccinated	2 (2.2)
Treatment regimen, *n* (%)	
<7.5 mg prednisone	39 (42.4)
>7.5 mg prednisone	18 (19.6)
IS drugs	37 (40.2)
Hydroxychloroquine	79 (85.9)
SLEDAI, mean ± SD	3.33 ± 2.73
Laboratory findings	
dsDNAn levels, median (IQR)	14.50 (4.77–44.75)
C3 mg/dL, mean ± SD	96.97 ± 21.45
C4 mg/dL, mean ± SD	16.50 ± 7.64
Lymphocyte cells/uL, mean ± SD	1527.65 ± 585.26
CD4 cells/uL, ±SD	644.82 ± 283.29
CD4 (%)	
≤200	2.3
200–500	26.1
≥500	71.6
CD8 cells/uL, ±SD	495.67 ± 256.89
B cells/uL, median (IQR)	119.80 (65.35–215.40)
NK cells/uL, median (IQR)	167.50 (110.50–229.90)

**Table 2:** Results of TST and T.SPOT.TB.

Results of TST	Results of T.SPOT.TB			
	Negative	Positive	Indeterminate	Total
Negative	79	3	4	86
Positive	4	2	0	6

Total	83	5	4	92

**Table 3:** Correlation between TST and T.SPOT.TB tests.

SLE patients	Kappa value
All patients	0.324
Patients not receiving IS/CTC	0.436
Patients receiving hydroxychloroquine	0.473

**Table 4:** TST positive *versus* TST negative patients. Univariate analyses.

Clinical and laboratory findings	TST positive (*n* = 6)	TST negative (*n* = 86)	*P* value
Age, years, mean ± SD	49.50 ± 14.69	42.23 ± 14.86	0.25
Patients with risk factors for LTBI, *n* (%)	2 (33)	7 (8)	0.10
SLE duration, median (IQR)	90 (21–237)	144 (60–225)	0.72
SLEDAI, mean ± SD	2.83 ± 3.37	3.37 ± 2.7	0.64
SLICC, median (IQR)	0 (0-1)	0 (0-1)	0.78
dsDNAn, UI/mL, median (IQR)	29 (2.45–61.25)	13.50 (4.77–42.75)	0.71
Prednisone > 7.5 mg/d, *n* (%)	0 (0)	18 (32)	0.68
Immunosuppressed patients, *n* (%)	1 (17)	58 (98.3)	0.021
Hydroxychloroquine treatment, *n* (%)	4 (66)	75 (87)	0.19
Steroid dose, mg, mean ± SD	0.83 ± 2.04	4.09 ± 4.92	0.11
Steroid cumulative dose, mg, mean ± SD	2275 ± 4056.32	19019.35 ± 22249.92	0.001
Cumulative steroids/disease duration, mg/year, mean ± SD	309.06 ± 742.74	1696.47 ± 1433.26	0.021
Mycophenolate dose, mg, mean ± SD	0	267.55 ± 538.20	0.001
Mycophenolate cumulative dose, mg, mean ± SD	0	643743.02 ± 1329836.44	0.001
Cumulative mycophenolate/disease duration, mg/year, mean ± SD	0	76986.98 ± 160990.33	0.001
Methotrexate dose, mg, mean ± SD	0	1.30 ± 3.33	0.001
Methotrexate cumulative dose, mg, mean ± SD	0	336.98 ± 801.21	0.001
Cumulative methotrexate/disease duration, mg/year, mean ± SD	0	49.35 ± 132.52	0.001

**Table 5:** T.SPOT.TB positive *versus* T.SPOT.TB negative patients. Univariate analyses.

Clinical and laboratory findings	T.SPOT positive (*n* = 5)	T.SPOT negative (*n* = 87)	*P* value
Age, years, mean ± SD	62.40 ± 12.75	41.57 ± 14.25	0.002
Patients with risk factors for LTBI, *n* (%)	1 (20)	8 (9.2)	0.41
SLE disease duration SLE, median (IQR)	174 (135–357)	126 (60–207)	0.10
SLEDAI, mean ± SD	2.4 ± 2.5	3.39 ± 2.75	0.43
SLICC, median (IQR)	0 (0–0.75)	0 (0-1)	0.53
dsDNAn, UI/mL, median (IQR)	13.70 (2.05–33)	14.50 (3.72–45.50)	0.65
Daily prednisone > 7.5 mg, *n* (%)	1 (20)	17 (19.5)	0.53
Immunosuppressed patients, *n* (%)	2 (40)	57 (65.5)	0.56
Hydroxychloroquine treatment, *n* (%)	4 (80)	75 (86.2)	0.54
Steroid dose, mg, mean ± SD	3 ± 4.47	3.93 ± 4.89	0.67
Steroid cumulative dose, mg, mean ± SD	18486 ± 18460.34	17895.22 ± 22196.28	0.94
Cumulative steroids/duration of disease, mg/year, mean ± SD	1076.41 ± 1099.36	1636.42 ± 1454.14	0.40
Mycophenolate dose, mg, mean ± SD	216 ± 482.99	252.06 ± 529.19	0.88
Mycophenolate cumulative dose, mg, mean ± SD	4800 ± 10733.12	636067.81 ± 1324014	0.001
Cumulative mycophenolate/disease duration, mg/year, mean ± SD	23.52 ± 52.61	76100.72 ± 160264.94	0.001
Methotrexate dose, mg, mean ± SD	0	1.29 ± 3.32	0.38
Methotrexate cumulative dose, mg, mean ± SD	216 ± 482.99	320.69 ± 794	0.77
Cumulative methotrexate/disease duration, mg/year, mean ± SD	1.05 ± 2.36	48.72 ± 131.88	0.42

**Table 6:** T.SPOT.TB indeterminate *versus* T.SPOT.TB determinate results. Univariate analyses.

Clinical and laboratory findings	Indeterminate T.SPOT.TB (*n* = 4)	Determinate T.SPOT.TB (*n* = 88)	*P* value
Age, years, mean ± SD	55.50 ± 13.17	42.13 ± 14.76	0.079
Patients with risk factors for LTBI, *n* (%)	1 (25)	8 (9)	0.34
SLE disease duration, median (IQR)	318 (117–351)	138 (60–207)	0.028
SLEDAI, mean ± SD	2.25 ± 1.25	3.38 ± 2.78	0.42
SLICC, median (IQR)	1 (1–1.75)	0 (0-1)	0.002
dsDNAn, UI/mL, median	13.5 (6.77–332.75)	14.50 (3.72–43.50)	0.85
Prednisone > 7.5 mg/d, *n* (%)	0 (0)	18 (33.3)	0.31
Immunosuppressed patients, *n* (%)	3 (75)	56 (63.6)	0.64
Hydroxychloroquine treatment, *n* (%)	2 (50)	77 (87.5)	0.94
Steroid dosage, mg, mean ± SD	2.62 ± 2.05	3.94 ± 4.93	0.59
Steroid cumulative dose, mg, mean ± SD	35745.62 ± 27062.79	17117.40 ± 21498.45	0.09
Steroid cumulative dose/disease duration, mg/year, mean ± SD	1766.47 ± 1248.49	1598.69 ± 1451.84	0.82
Mycophenolate dose, mg, mean ± SD	0	261.47 ± 533.49	0.001
Mycophenolate cumulative dose, mg, mean ± SD	0	629112.50 ± 1317998.72	0.001
Mycophenolate cumulative dose/disease duration, mg/year, mean ± SD	0	75237.27 ± 159546.95	0.001
Methotrexate dose, mg, mean ± SD	1.25 ± 2.5	1.22 ± 3.28	0.98
Methotrexate cumulative dose, mg, mean ± SD	150 ± 300	322.50 ± 793.78	0.66
Cumulative methotrexate dose/disease duration, mg/year, mean ± SD	13.63 ± 27.27	47.61 ± 131.30	0.60
